# P-149. Increased detection of Clostridioides difficile in adults’ stool specimens with multiplex infectious diarrhea panel nucleic-acid amplification testing

**DOI:** 10.1093/ofid/ofaf695.375

**Published:** 2026-01-11

**Authors:** Eugene Yeung

**Affiliations:** University of British Columbia

## Abstract

**Background:**

Since 2022, diagnostic laboratories in British Columbia (BC), Canada, have been advised to replace traditional bacterial, viral and parasitic intestinal pathogen testing with multiplex infectious diarrhea panel nucleic-acid amplification testing (IDP-NAAT), which contains a minimum of 14 common pathogens, including *C. difficile*. However, multiplex testing can lead to false positive targets which are not even in the clinicians’ differentials. The Infectious Diseases Society of America Guidelines (2017) for *C. difficile* infection have specific indications for testing rather than routine testing for all patients with diarrhea. The current study investigated whether multiplex IDP-NAAT would lead to higher prevalence of positive *C. difficile* results, signaling a pseudo-outbreak.

**Methods:**

LifeLabs BC microbiology laboratories, connected with 129 collection centres in communities in the province, provided the laboratory data on *C. difficile* results from adult patients (age >18 years). An audit was conducted from September 2022 to August 2023, one year prior to implementation of IDP-NAAT in LifeLabs, when *C. difficile* NAAT followed with antigen testing was used, per request only. Another audit was conducted from October 2023 to September 2024, one year after the implementation of multiplex IDP-NAAT followed with *C. difficile* antigen testing for confirmation. Chi-square with Yates correction was used to compare the change.

**Results:**

Prior to implementation of IDP-NAAT, 1475 of 21912 patient’s stool specimens (6.73%) submitted for testing was positive for *C. difficile*. After the implementation, 2838 of 56590 patients’ stool specimens (5.02%; *p* < 0.05 vs. prior) submitted for testing was positive for *C. difficile*.

**Conclusion:**

Although the positivity rate of *C. difficile* decreased, the number of positive *C. difficile* results increased almost twice after the implementation of IDP. This increase could mislead epidemiologists to suspect an outbreak and clinicians to overprescribe for *C. difficile* colonization rather than infection. More communications and educations may be warranted to remind clinicians that positive *C. difficile* NAAT results are not always indicative of infection due to implementation of multiplex testing on all stool specimens.

**Disclosures:**

All Authors: No reported disclosures

